# Supplementary material for: Patterns of Intron Gain and Loss in Fungi
Source: PLoS Biol. 2004 Nov 30;2(12):e422. doi: 10.1371/journal.pbio.0020422 (PMC532390; doi:10.1371/journal.pbio.0020422)
Supplement: Table S1 — Also available at http://genes.mit.edu/NielsenEtAl/. (4.3 MB ZIP). [file pbio.0020422.st001.zip › NielsenEtAl/html/630.html]

AN1254.1.NCU03206.1.MG01249.1.FG00477.1


```
 CLUSTAL W (1.82) Multiple Sequence Alignments - Introns Inserted


Sequence 1: NCU03206.1	362 aa
Sequence 2: FG00477.1	370 aa
Sequence 3: MG01249.1	385 aa
Sequence 4: AN1254.1	377 aa
Alignment Length: 403 aa
Number Identitical Residues: 114 aa
Alignment Score (without introns) 5873


MG01249.1 	MAKATSPPD-SPLSSMDESDAF----NEDGHDTPDP-MPPAKRQKLQLDVSTASSAVAGP
NCU03206.1	MTREHSPADDSPLSSMASSDEEEIFADEVPEAVDEPEEPPTKRIKVTSGSTASSAVIQEP
FG00477.1 	MARDSSPPE-SPLSSTYESEID----EDGGHDYDESTLRPSKRQRLEAGSTTSSAVVPDA
AN1254.1  	MAD--SPGS--PLSSVASDDMSDRDDPKQGVSTPSSNMPPSKRRRTGMASWDRNTPVSTS
          	*:   ** .  ****  ..:  .    .      ..   *:** :        .: :  .

MG01249.1 	D----PNDPALEGLSPVSSDTSGDIPSSPAHHPHHPHHLRHGDTDDDLH----EQVSVCA
NCU03206.1	E----ADPDFPDGMSDVSSDTDGDIPSSPINARQD---------EDDFQ----EQVTVCA
FG00477.1 	EPEAVPEPDPLEGMSDVSSDTSGDIPSSPINARLD---------EEDFQ----EQVTKCD
AN1254.1  	FQDELPPPPSPS--SSISSDTSGEIPNSPNTLSLIG-----ANQDEDYSGFCNDQVTVCR
          	  .  .     .  * :****.*:**.**            .. ::*  . ..:**: * 

MG01249.1 	WGDCDAGDLGNMDRLVEHIHSDHIEGKQKNNYICEWKGCGRNRQLHASGYALKAHMRSHT
NCU03206.1	WEGCKVGDLGNMDRLVEHIHNSHIEGRQKK-YTCEWIGCSRKSLPHASGYALKAHMRSHT
FG00477.1 	WDGCPAGDQGNMDKLVEHIHNSHIENRQKK-YTCEWMSCSRKGLPHASGYALKAHMRSHT
AN1254.1  	WEGCDFKDLGNMDDLVQHIHNEHVGSRQKK-YSCEWSDCTRKGQTHASGYALRAHMRSHT
          	* .*   * **** **:***..*: .:**: * *** .* *:   *******:*******

MG01249.1 	REKPFYCYLPE1CDRSFTRSDALAKHMRTVHETEALRPSDPIPK--AMQAQTAAGTSTKS
NCU03206.1	REKPFYCYLPE1CDRAFTRSDALAKHMRTVHETEALRPSDPVPK--SMQAGG--GPAGKS
FG00477.1 	REKPFYCYLPE1CDRSFTRSDALAKHMRTVHETEALRPSDPVPK--SMQSG----PQGKG
AN1254.1  	REKPFYCALPE1CDRSFTRSDALAKHMRTVHETEALRPSDPVPKRHDILSSGGTPVGTPA
          	******* *** ***:*************************:**   : :  .:     .

MG01249.1 	GKPLTIVIKTPRSHAAGARDSVLDRDGASPGSENSFQFTE--LTSEQGFSPKELAMPLPK
NCU03206.1	AK-VKIIIRRPDSHAAGQDDSVDDAS----GGDDGADFTP--LTEDHGFTTKELDMPLDR
FG00477.1 	GK-IKIIMKT-NQHSQGGHDDTDEIN----GDEANAEFFT--PLTDEIFTLDELQLPVDK
AN1254.1  	SKLQRIKLKLSHPPRDDSEQYSEGVNDDTAGTEDLDEFEVPEFSSDTGFDDHELEMNPHQ
          	.*   * :: .     .  :     .. :.* :   :*  ..   :  *  .** :   :

MG01249.1 	LHNLLRCTLKWTEEEQQELQREIEAAETAYKNAWLEKEVLLHQVIQNEINWHERRQAVLS
NCU03206.1	LMKLCRLQVKLVEAEGEKLRKDCKLWEELYKQEWLEKEILLDQVVRSEQAWHARRQAVLK
FG00477.1 	LYRKCHWEAKWAEEIGESLAKECKEWENIYFQEWREKEVLVHQAIKSEINWHDRRQAILT
AN1254.1  	LYRLLRRQIHWAEKEGADLKSTWEKIRPKRKQAWLEKEAIFDDLMDAELRLLSTLVSAEG
          	* .  :   : .*    .*    :  .    : * *** :..: :  *        :   

MG01249.1 	GVADVQAKPVAVNGTPAAAATAAADDADSVGGLTEEEMETTMQD
NCU03206.1	GMADVQLP---------EKAVANGTAGEN-DTAEA--ARLTVEE
FG00477.1 	GAVDVQVPGAVESVESVEGEKTNGAQYEDQDTATNGIAASEIEE
AN1254.1  	ATGQTSVVP------------ANGMPENPAHPIAA--AGYQIPP
          	.  :..               : .   :             :
```
